# Ice Nucleation Mechanisms on Platinum Surfaces in PEM Fuel Cells: Effects of Surface Morphology and Wettability

**DOI:** 10.1002/advs.202406861

**Published:** 2024-08-08

**Authors:** Jiaqi Wang, Linhao Fan, Lincai Li, Qing Du, Kui Jiao

**Affiliations:** ^1^ State Key Laboratory of Engines Tianjin University 135 Yaguan Road Tianjin 300350 China; ^2^ National Industry‐Education Platform for Energy Storage Tianjin University 135 Yaguan Road Tianjin 300350 China

**Keywords:** catalyst surface morphology, cold start, heterogeneous ice nucleation, molecular dynamics simulation, proton exchange membrane fuel cell

## Abstract

Understanding the ice nucleation mechanism in the catalyst layers (CLs) of proton exchange membrane (PEM) fuel cells and inhibiting icing by designing the CLs can optimize the cold start strategies, which can enhance the performance of PEM fuel cells. Herein, mitigating the structural matching and templating effects by adjusting the surface morphology and wettability can inhibit icing on the platinum (Pt) catalyst surface effectively. The Pt(211) surface can inhibit icing because the atomic spacing of (211) crystalline surface is much larger than the characteristic distance of ice crystal, thereby mitigating the structural matching effects. A water overlayer on the Pt surface induced by the strong attraction of Pt can act as a template for ice layers and plays an important role in the icing process. Buckling of water overlayer due to the larger atomic spacing of (211) crystalline surface mitigates the templating effect and inhibits icing. Moreover, the water overlayer on the hydrophobic Pt(211) surface with fewer water molecules also mitigates the templating effect, which makes ice nucleation more difficult than homogeneous nucleation. These findings reveal the ice nucleation mechanisms on the Pt catalyst surface from the molecular level and are valuable for catalyst designs to inhibit icing in CL.

## Graphical abstract

A water overlayer on the Pt surface induced by the strong attraction of Pt atoms can act as a template for the ice layers and plays an important role in the ice nucleation process. Mitigating the structural matching and templating effects by adjusting the surface morphology and wettability can inhibit icing on the Pt catalyst surface during the cold start.

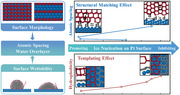

## 1 Introduction

Owing to their high energy conversion efficiency, fast start‐up, high energy density, and low pollution, proton exchange membrane fuel cells (PEMFCs) show potential in energy storage and automotive applications.^[^ ^1^, ^2^, ^3^ ^]^ The process of PEMFC starting from the low‐temperature environment until stabilizing the output performance is called the cold start. The water generated by the electrochemical reaction in the cathode catalyst layers (CLs) can freeze in the porous structure and even in the flow channels of the PEMFC at low temperatures.^[^ ^4^, ^5^ ^]^ The accumulation of ice in the CL can block the gas transport pathway and cover the catalyst reaction sites, leading to a failed cold start.^[^ ^6^ ^]^ Moreover, ice can cause worm and buckling deformations of CL, inducing PEMFC degradation. Therefore, understanding the mechanism of ice nucleation in CL is critical for achieving a successful cold start and sustaining the high performance of PEMFC at low temperatures.

To elucidate the icing process and its influences inside the PEMFC during the cold start, many researchers have simulated the cold start process of PEMFCs.^[^ ^7^, ^8^, ^9^, ^10^ ^]^ Wang et al.^[^ ^11^ ^]^ established a lumped parameter model to study ice formation during cold start and found that the reduction of the effective reactive area in the CL due to ice coverage was the main reason for voltage loss. Balliet et al.^[^ ^12^ ^]^ employed a 2D non‐isothermal transient model along the vertical membrane and vertical runner direction to study the distribution of supercooled water and ice during the cold start and found that the distribution of supercooled water has an important effect on ice melting and drainage after a successful cold start. Gwak et al.^[^ ^13^ ^]^ built a numerical model to study the icing–melting process in CL and found that the water content increases in the melting stage because the electrolyte absorbs the melted ice. These models can investigate the effects of icing on PEMFC performance at the macroscopic scale, ignoring the underlying icing mechanisms in CLs. Furthermore, studies have focused on cold start strategies, such as gas heating and internal catalytic hydrogen–oxygen reactions.^[^ ^4^, ^5^ ^]^ However, understanding the icing mechanisms further and proposing the design of CL to inhibit icing are of significance for optimizing the cold start strategies, which can enhance the performance of PEMFC.

Experimental studies have been conducted to observe ice nucleation in the CL via transmission electron microscopy (TEM) and neutron imaging.^[^ ^14^, ^15^ ^]^ However, TEM struggles to provide molecular‐level information regarding individual ice nucleation events, while neutron imaging, depending on the reactor, can be costly. Capturing the phenomenon of icing using experiments that consider nanometer‐sized catalysts in CLs is challenging. Therefore, molecular dynamics (MD) simulations have been widely employed to understand ice nucleation processes. Several studies on MD have successfully observed heterogeneous ice nucleation in the past few years.^[^ ^16^ ^]^ B. Glatz et. al^[^ ^17^ ^]^ found that hydrogen bonding was necessary but not sufficient for observing ice nucleation on the surface. L. Lupi and his colleagues^[^ ^18^ ^]^ found that surfaces such as the graphite surface that promoted ice nucleation induced the layering phenomenon in the interfacial water, suggesting that the order of degree in the arrangement of surface atoms may play an important role in the mechanism of heterogeneous ice nucleation. Physically, ice nucleation is a rare event that requires a long‐time simulation, which poses a considerable challenge to the computational cost of MD simulations. Therefore, various methods have been embedded into MD simulations to calculate the free energy barriers and ice nucleation rates, such as forward flux sampling,^[^ ^19^ ^]^ metadynamics,^[^ ^20^ ^]^ and umbrella sampling.^[^ ^21^ ^]^

**Figure 1:**
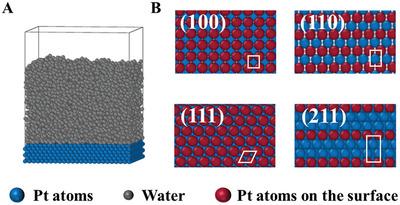
A) Simulation system used for ice nucleation on Pt surfaces. The coarse‐grained model of water (mW) is used in the model. The mW molecules are shown as gray spheres, while the Pt atoms are blue. A 2 nm vacuum layer is placed on top of the simulation box to avoid the effect of periodicity along the water film thickness direction on ice nucleation B) Top views of four types of Pt crystalline surfaces. The Pt atoms on the surface are shown as red spheres.

Platinum (Pt) is a widely used catalyst in PEMFCs. For Pt‐based nanocatalysts in PEMFCs, researchers have found that the atomic arrangement on the crystalline surface significantly affects the catalytic activity and selectivity.^[^ ^22^, ^23^ ^]^ Pt (111), (100), (110), and (211) crystalline surfaces have been widely used in CLs of PEM fuel cells and show superior performance.^[^ ^24^, ^25^ ^]^ Specifically, the Pt(111) crystalline surface demonstrates a higher surface energy and catalytic activity for the oxygen reduction reaction (ORR) compared to the Pt(100) crystalline surface. However, the selective adsorption of oxygen on the Pt(100) crystalline surface surpasses that on the Pt(111) crystalline surface.^[^ ^24^ ^]^ Previous works have shown that the ORR activity of Pt(211) crystalline surface is similar to that of Pt(111) crystalline surface.^[^ ^25^ ^]^ Although numerous studies have explored the impact of Pt crystal faces on catalytic activity, their effects on icing at low temperatures have not been explored. Therefore, we utilize MD simulations to investigate the ice nucleation process on the Pt (111), (100), (110), and (211) crystalline surfaces, taking into account the influence of wettability. Four Pt crystalline surfaces have significant differences in the atomic separation and symmetry of the first crystalline layer, as shown in **Figure** **1**. This study aims to provide insights into the ice nucleation mechanisms and guide the mitigation of icing during the cold start of PEMFCs through CL designs.

## 2 Results and Discussion

### 2.1 Ice Nucleation Process on Pt Surfaces

**Figure 2:**
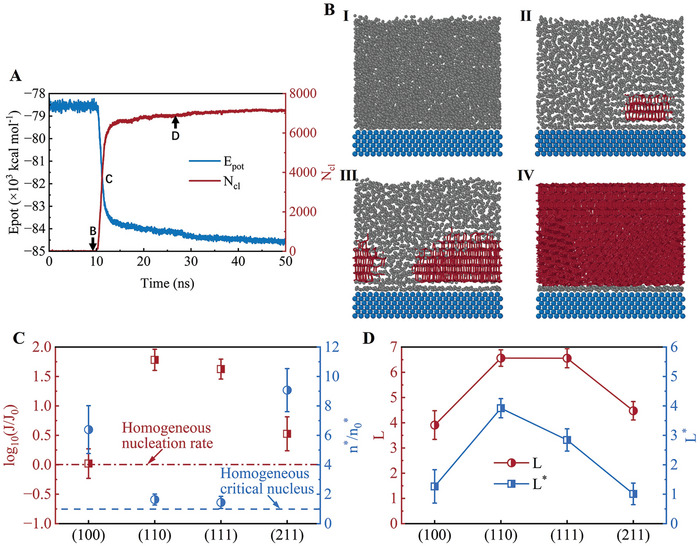
Ice nucleation process of supercooled water on Pt(100) surface. A) Change in the potential energy of water molecules (*E*_pot_) and the number of water molecules in the largest ice cluster (*N*_cl_). B) Different stages of ice cluster formation during simulated ice crystallization. The ice cluster is represented by red rods, while the mW molecules are represented by gray balls. C) Ice nucleation rates and critical nuclei size on Pt surfaces. The dashed line represents the ice nucleation of pure water. D) Layering *L* (red) and layering excluding the water overlayer L^*^ (blue) of water on Pt surfaces.

First, the processes of ice nucleation on different Pt crystalline surfaces are discussed. The change in the potential energy of water molecules (*E*_pot_) and the number of water molecules in the largest ice cluster (*N*_cl_) on Pt(100) surface over time is calculated, as shown in **Figure** **2**A. Snapshots of the nucleation and growth of water molecules on the Pt(100) surface along the typical crystallization trajectory are shown in Figure 2B I–IV. The processes of ice nucleation on Pt(110), Pt(111), and Pt(211) are shown in Figures S1–S3 (Supporting Information), respectively. Initially, all critical nuclei on the four Pt crystalline surfaces appear from the interface between Pt and water (Stage II). Then, the ice nuclei begin to grow (Stage III) until they are completely frozen (Stage IV). As shown in Figure 2A, the time from the beginning to the appearance of critical nuclei is referred to as the icing induction time. The results show that different Pt crystalline surfaces lead to different ice nucleation processes and thus different induction times. The icing induction time on the Pt(211) surface is the longest, which demonstrates that ice nucleation on the Pt(211) surface is more difficult than that on other crystalline surfaces.

### 2.2 Ice Nucleation Rates and Layering of Water on Pt Surface

The ice nucleation rates and the sizes of critical nuclei on the four Pt crystalline surfaces are calculated and compared with homogeneous nucleation. As shown in Figure 2C, the ice nucleation rates on the four Pt crystalline surfaces are higher than the homogeneous nucleation rate, which suggests that Pt can promote icing and decrease the ice nucleation energy barrier. Furthermore, the results show that the ice nucleation rate is inversely proportional to the critical nucleus size. A lower ice nucleation rate and larger critical nucleus can make icing in CLs more difficult. The largest critical nucleus is present on the Pt(211) surface, indicating that icing on the Pt(211) surface is more difficult. Moreover, the icing induction time on the Pt(211) surface is the longest, as shown in Figure S3 (Supporting Information). Furthermore, the Pt with (211) crystalline surface has a high catalytic efficiency.^[^ ^25^ ^]^ Therefore, the Pt with (211) crystalline surface with a high ORR activity is preferred to effectively inhibit icing during the cold start. The direct one‐pot synthesis with homogeneous nucleation and galvanic replacement methods could be used to generate Pt in various morphologies, exposing with low‐ or high‐index crystalline surfaces.^[^ ^26^ ^]^

To explain the trend of the ice nucleation rate, the layering *L* of water on the Pt surface is calculated as follows:^[^ ^18^ ^]^

(1) $$L=\int_{0}^{z_{bulk}}{\left|\frac{\rho (z)}{\rho_{0}}-1\right|}^{2}dz$$

where the *z_bulk_* is the height above the surface at which the water density converges to ρ_0_, the ρ(*z*) is the local water density at a height *z* above the Pt surface, and the ρ_0_ is the density of bulk liquid water. However, due to its high adsorption energies, the layering excluding the contact layer L^*^ on the Pt surface should be calculated as follows:^[^ ^27^ ^]^

(2) $$L^{*}=\int_{z_{0}}^{z_{bulk}}{\left|\frac{\rho (z)}{\rho_{0}}-1\right|}^{2}dz$$

where the *z*_0_ is a height so that the layering contributions of the contact layer are excluded. Herein, the *z_bulk_* is 1.5 nm, as shown in Figure S4 (Supporting Information). The layering *L* and L^*^ of water on the Pt surface is highly correlated with their freezing efficiency. As shown in Figure 2D, the smaller *L* and L^*^ values of water lead to a lower ice nucleation rate. *L* and L^*^ reflect the change in the adsorption structure of the water molecules on the Pt surface. Larger *L* and L^*^ represents a higher ordering of water molecules on the Pt surface before icing. Furthermore, the results show that the atomic spacing on the Pt surface can affect *L* and L^*^. The dense arrangement of Pt atoms on the Pt(111) surface leads to a larger L^*^, which means that icing on the Pt(111) goes through a shorter pathway and overcomes a lower nucleation barrier. The smaller L^*^ resulted from the large Pt atom spacing on the Pt(211) surface, leading to a lower nucleation rate and longer icing induction time. Therefore, reasonable atomic spacing can effectively inhibit icing on the Pt catalyst surface during a cold start.

### 2.3 Templating and Structural Matching Effects

**Figure 3:**
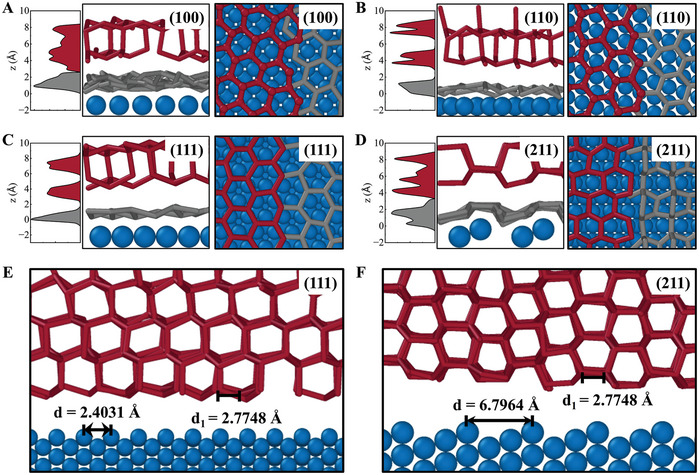
Number density, side view, and top view of mW molecules on the A) Pt(100), B) Pt(110), C) Pt(111), and D) Pt(211) surfaces after freezing. E) Structural match between ice and Pt(111) surface. F) Structural match between ice and Pt(211) surface. *d*_1_ is the characteristic distance of ice, and *d* is the atomic spacing of the Pt surface. In all cases, the higher layers are red, while the contact layer is gray.

The number density distribution of water molecules along the through‐plane direction is calculated and compared with the visualization results, as shown in **Figure** **3**A–D. The results show that the water overlayer is formed on the Pt surface during icing owing to the strong interaction between Pt and water. Although the interaction between Pt and water is the same, the structures of the water overlayer are different on the four Pt crystalline surfaces. At the same time, the structure of the water overlayer is hexagonal, which can act as a template for the ice layers on it and thus plays an important role in the ice nucleation process, as shown in Figure 3A–D. However, the water overlayer on the Pt(211) surface is a bilayer structure because the distance between the atoms on the Pt surface is so large that the second layer of Pt atoms can adsorb the water molecules, as shown in Figure 3D. Buckling of the water overlayer mitigates the templating effect of ice nucleation, resulting in the largest critical nuclei on the Pt(211) surface. These results suggest that the ration atomic arrangement on Pt surfaces can effectively inhibit ice nucleation. Moreover, the water overlayer on the Pt surfaces may protect the catalyst structure from damage, thus prolonging the lifetime of the PEMFCs. The hexagonal structure of the water overlayer can allow gas to pass through and react on the catalyst surface, thereby having minimal impact on gas transport. Figure 3E,F demonstrate the characteristic distance (*d*_1_) of ice compared with the atomic spacing (*d*) of the Pt (111) and Pt (211) surfaces. The atomic spacing can be modified by adjusting the crystalline surface of Pt. The ice nucleation on the Pt surface is significantly accelerated when the structure of the Pt surface is well matched to the structure of the ice shown in Figure 3E, namely, the structural matching effect. The results show that *d* is much larger than *d*_1_, which indicates that the low structural matching effect between Pt(211) and ice can effectively inhibit icing. Simultaneously, this leads to the inability of the Pt(211) surface to accommodate the hexagonal overlayer template, as shown in Figure 3F. In summary, a larger atomic spacing and unordered water overlayer on the Pt(211) surface mitigate the structural matching and templating effects, which can inhibit icing on the Pt(211) surface effectively.

### 2.4 Ice Crystal Morphology on Pt Surface

**Figure 4:**
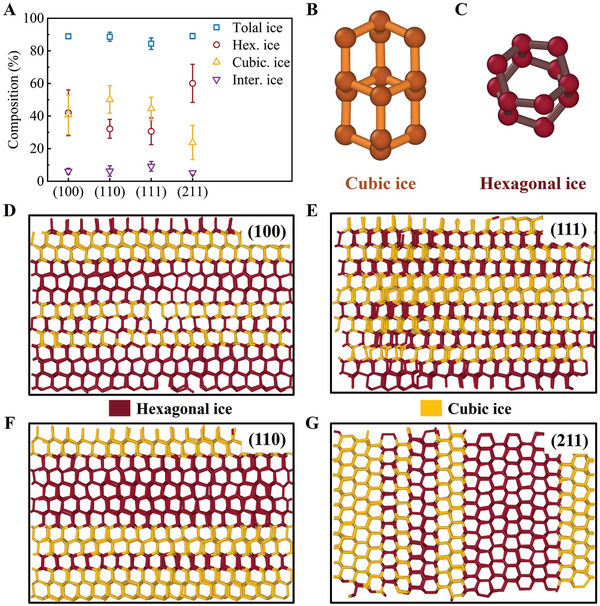
A) Composition of total ice‐like, hexagonal ice, cubic ice, and interfacial ice molecules on different Pt surfaces. Units of B) cubic ice and C) hexagonal ice. Comparison of the distribution of icing cubic ice and hexagonal ice on the (D) Pt(100), (E) Pt(110), (F) Pt(111), and (G) Pt(211) surfaces.

To understand the crystallization mechanism of ice nucleation, the structures of ice formed on Pt surfaces have been quantified. The CHILL algorithm^[^ ^28^ ^]^ is employed to identify cubic ice (*I*_c_) and hexagonal ice (*I*_h_) on the Pt surface. **Figure** **4**A shows the composition of the total ice‐like, *I*_h_, *I*_c_, and interfacial ice molecules on different Pt surfaces. The results show that the content of ice‐like molecules makes up ≈90% of all water molecules on the four Pt surfaces, which proves that the Pt surface is covered by the water overlayer during icing. The contents of *I*_c_ and *I*_h_ are irrelevant to the rate of freezing and only affect the structure of the ice crystals. The contents of *I*_c_ and *I*_h_ on the Pt(100) surface are similar, while the amount of *I*_c_ is larger than that of *I*_h_ after icing on the Pt(211) surface. The thermodynamic stability of *I*_h_ is higher than that of *I*_c_.^[^ ^29^ ^]^ Therefore, the thermodynamic properties of the ice crystals on the Pt(211) surface are stable and less easily destroyed.

Figure 4B,C shows the units of *I*_c_ and *I*_h_, respectively. Figure 4D–G illustrates the distribution of *I*_c_ and *I*_h_ on different Pt surfaces. The heterogeneous ice crystal structure on the Pt surface comprises a disordered ice state with a mixture of *I*_c_ and *I*_h_ instead of generating a single ice crystal structure. To compare the effects of the Pt surface on ice ordering, the *I*_c_ and *I*_h_ distributions of pure water are analyzed, as shown in Figure S5 (Supporting Information). The results show that Pt can induce ice layering. Meanwhile, the layering of water on the surface of Pt (211) shows an in‐plane layering structure, which is obviously different from those on the other three surfaces. The sparse arrangement of the Pt atoms in the first layer on the surface of Pt (211) gives the water molecules enough space to make contact with the Pt atoms in the second layer, which induces the formation of in‐plane layering.

### 2.5 Adjusting Surface Wettability to Inhibit Icing

**Figure 5:**
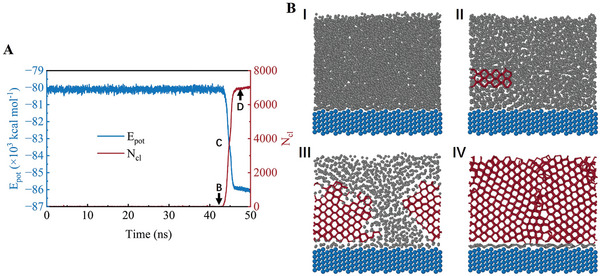
Ice nucleation process of supercooled water on hydrophobic Pt(211) surface. A) Change in the potential energy of water molecules (*E*_pot_) and number of water molecules in the largest ice cluster (*N*_cl_). B) Different stages of ice cluster formation during simulated ice crystallization. The ice cluster is represented by red rods, and the liquid water molecules are represented by gray balls.

**Figure 6:**
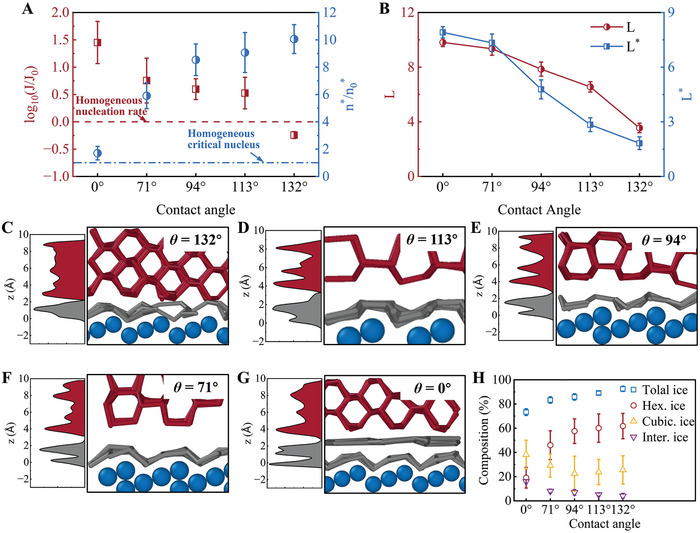
A) Ice nucleation rates and critical nuclei sizes on Pt(211) surfaces. The dashed line represents the ice nucleation of pure water. B) Layering *L* (red) and layering excluding the water overlayer L^*^ (blue) of water on Pt(211) surfaces. Number densities and side views of mW molecules on the Pt(211) surfaces with contact angles of C) 132°, D) 113°, E) 94°, F) 71°, and G) 0° after freezing. In all cases, the water layer close to the Pt surface is represented by gray bonds, while the other water molecules are represented by red bonds. H) Composition of total ice‐like, hexagonal ice, cubic ice, and interfacial ice molecules on Pt(211) surfaces with different contact angles.

Mitigation of the structural matching and templating effects is found to inhibit icing by changing the Pt surface morphology. To further mitigate the template effect, five Pt(211) surfaces with different wettabilities are constructed by altering the *ε* parameter in the L–J potential. The contact angles are calculated as 132°, 113°, 94°, 71°, and 0°, respectively, as shown in Figure S6 (Supporting Information). Notably, the contact angles are calculated using potentials that are the same as those in previous work,^[^ ^30^ ^]^ which verified the reliability of the model. The process of ice nucleation on a hydrophobic surface, as shown in **Figure** **5**, suggests that the critical nucleus is generated inside water instead of the interface between water and Pt. As shown in Figure 5A, the icing induction time of the hydrophobic surface is longer than that of the other surfaces. This demonstrates that the hydrophobic surface can further inhibit ice nucleation. Meanwhile, the ice nucleation rate and critical nucleus size are also calculated, as shown in **Figure** **6**A. The results show that the ice nucleation rate increases and the critical nucleus size decreases with increasing Pt surface wettability, which proves that the hydrophobic surface can inhibit icing on the Pt surface. In particular, the ice nucleation rate is even lower than that of homogeneous nucleation when the contact angle is 132°. The layering *L* and L^*^ of mW molecules on the Pt surface before icing are calculated, as displayed in Figure 6B. Both *L* and L^*^ decrease as the hydrophobicity of the Pt surface increases, demonstrating that water molecules are less ordered on the hydrophobic Pt surface and that the energy barrier of ice nucleation increases. The adsorption of water molecules is weakened because of the reduced interaction between Pt and water on the hydrophobic surface, which mitigates the templating effect of ice nucleation. Therefore, the hydrophobic surface can effectively inhibit icing on the Pt surface.

Similarly, the number density distribution of water molecules on the Pt surface shown in Figure 6C–G indicates that the thickness of the water overlayer increases with increasing wettability because the enhanced interaction between Pt and water results in more water molecules being adsorbed onto the Pt surface, particularly for the superhydrophilic surfaces shown in Figure 6G. Although the thicknesses of the water overlayers on Pt surfaces with contact angles of 132°, 113°, 94°, and 71° do not significantly increase, the numbers of water molecules inside the water overlayers increase. As shown in Figure S7 (Supporting Information), fewer water molecules inside the water overlayer mitigate the template effects, resulting in a lower ice nucleation rate. To clearly illustrate this phenomenon, the composition of the ice crystals is calculated and displayed in Figure 6H. The results show that the ice content decreases as wettability increases. Although icing is more challenging on the hydrophobic surface, the lower amount of ice on the hydrophilic surface means that less heat production is required to melt, resulting in an easy cold start. Moreover, the decreasing content of *I*_h_ and the increasing content of *I*_c_ demonstrate that the structure of ice on the Pt surface is more unstable. Furthermore, previous works have modulated the surface wettability of catalysts by regulating the potential without changing the surface morphology of catalysts,^[^ ^31^ ^]^ which can prove that the surface wettability can be changed via an experiment to inhibit icing.

## 3 Conclusion

Herein, the ice nucleation processes and mechanisms on Pt catalyst surfaces with different morphologies and wettabilities are investigated using MD simulations. The atomic spacing of the Pt(211) surface is larger than the characteristic distance of ice, resulting in the longest ice induction time and largest critical nucleus because of the weak structural matching effect between the ice and Pt(211) surface. The water overlayer formed on the Pt surface can act as a template for icing and protect the structure of the Pt catalyst from being destroyed. The buckling of the water overlayer on the Pt(211) surface mitigates the templating effect of ice nucleation, resulting in a lower ice nucleation rate on the Pt(211) surface. Moreover, the results show that the percentage of *I*_h_ on the Pt(211) crystalline surface is higher than that of *I*_c_; thus, the ice thermodynamic properties are more stable. The heterogeneous ice crystal structure on the Pt surface comprises a disordered ice state with a mixture of *I*_c_ and *I*_h_ instead of generating a single ice crystal structure. The ice nucleation rate decreases and the critical nucleus size increases as surface wettability decreases. Therefore, the hydrophobic surface can inhibit icing because of the lower‐order degree of water on the surface. Furthermore, the thickness of the water overlayer decreases with decreasing wettability, which mitigates the templating effect, resulting in a lower ice nucleation rate on the hydrophobic surface. The percentage of *I*_c_ increases with increasing surface wettability, resulting in an unstable ice structure. These findings suggest that the Pt(211) catalyst with a hydrophobic surface can inhibit ice nucleation, which is valuable for catalyst designs to achieve a perfect cold start.

## 4 Experimental Section

### Simulation System

A Pt flat plate model with crystalline surfaces covered with a water film with ≈7000 water molecules was constructed, and the thickness of the water film is 5 nm. The effects of icing at the interface between water and Pt deserve more attention. Owing to the periodicity of different Pt crystalline surfaces, maintaining the same simulation box size for all simulations was impossible. The sizes of all simulation boxes were approximately kept as 65 Å × 65 Å × 80 Å. A 2 nm vacuum layer is placed on top of the simulation box to avoid the effect of periodicity along the water film thickness direction on ice nucleation.

### Force Field

The interactions between water molecules are described by the coarse‐grained model of water.^[^ ^32^ ^]^ The mW model with high computational efficiency represents each water molecule as a single particle forming a tetrahedral “hydrogen‐bonded” structure through three‐body nonbonding interactions that have no hydrogen atoms and electrostatic charges.^[^ ^33^ ^]^ Interactions between water molecules contain two‐ and three‐body interactions, as described by the functional form of the Stilinger–Weber potential. The mW water model can reproduce the structures of liquid water and ice and the transitions between them, and it is widely employed to study ice nucleation.^[^ ^16^, ^17^ ^]^ The interaction between water and Pt uses the Lennard–Jones potential:

(3) $$U\left(r\right)=\left\{\begin{array}{cc} 4\varepsilon \left[{\left(\frac{\sigma }{r}\right)}^{12}-{\left(\frac{\sigma }{r}\right)}^{6}\right] & r<r_{c}\\ 0 & r\geq r_{c} \end{array}\right.$$

where *r* is the distance between the mW water and Pt atom. The cutoff distance is set to *r_c_* = 12 Å. The interaction between water molecules and Pt in the simulation is *ε* = 0.2958 kcal mol^−1^ and *σ* = 0.2466 nm, resulting in a contact angle of 115°.^[^ ^30^ ^]^ In this simulation, the contact angle is calculated as 113°, which is consistent with previous work.^[^ ^30^ ^]^ Because the Pt surface is immobilized, the interaction between the Pt atoms is not defined.

### Simulation Procedures

The LAMMPS package was used for all MD simulations.^[^ ^34^ ^]^ Periodic boundary conditions were used in the simulations. The initial temperature for all simulations is set to 300 K. The NVT simulation is employed to allow the system temperature to vary four times at 300–280 K to remove the initial bias and relax the original configuration. The system coordinates are output every 0.5 ns after the system reaches equilibrium, and 20 coordinate files are collected. Several simulations with different initial coordinates were performed under the same conditions to eliminate the effect of initial randomness. The freezing temperature of all simulations was set to 210 K to ensure adequate icing. One case needs a 760 h calculation using 56 parallel CPU cores (Intel Xeon E5‐2680 v2, 2.80 GHz).

### Ice‐Like Molecule Identification

To accurately identify ice‐like molecules, the water molecule order parameter *Q_6_* was first calculated by simulating trajectories.^[^ ^28^ ^]^

(4) $$Q_{6m}\left(i\right)=\frac{\sum_{j}\sigma (r_{ij})Y_{6m}\left(r_{ij}\right)}{\sum_{j}\sigma (r_{ij})}$$

where *Y_6m_* is the sixth‐order spherical harmonic, *m* is a number from −6 to +6, and the function *σ*(*r_ij_*) is the switching function acting on the distance between atoms *i* and *j*. The parameters of the switching function are set such that the function is equal to 1 when atom *j* is located in the first coordination sphere of atom *i* and 0 otherwise. These vectors describe the degree of order of the coordination spheres around the mW molecules. The local order degree *q_6_* around the calculated mW molecule is later calculated by *Q_6_*.

(5) $$q_{6i}=\frac{\sum_{j}\sigma \left(r_{ij}\right)\sum_{m=-6}^{6}Q_{6m}^{*}\left(i\right)Q_{6m}^{*}\left(j\right)}{\sum_{j}\sigma (r_{ij})}$$

where *Q_6m_* (*i*) and *Q_6m_* (*j*) are the sixth‐order order parameters calculated for atoms *i* and *j*, respectively, and asterisks indicate complex conjugation. As shown in Figure S8 (Supporting Information), a clear distinction is found between ice and supercooled water at a cutoff value of 0.58. Therefore, the largest ice cluster is identified using *q_6_* > 0.58. The precise identification of ice is achieved by recognizing connected clusters found using a clustering algorithm and creating a new cluster containing atoms in the cluster and atoms within the cutoff of the cluster.^[^ ^35^ ^]^

### Ice Nucleation Rates

The mean first passage time (MFPT) method proposed by Wedekind et al^[^ ^36^ ^]^ was employed to evaluate the ice nucleation rate (J) on different Pt surfaces. The MFPT method has been successfully applied to many nucleation studies because of its inherent ability to directly provide the nucleation time, critical nucleus size, and nucleation barrier location by fitting the MFPT curve with the following expression:

(6) $$\tau \left(n\right)=\frac{\tau_{J}}{2}\left\{1+erf\left[\left(n-n^{*}\right)c\right]\right\}$$

where τ_*J*_ and n^*^ are the critical nucleation time and critical nucleus size, respectively. Parameter *c* is a constant associated with the Zeldovich factor *Z*, $c=Z\sqrt{\pi }$. The nucleation time for each cluster of size *n* is obtained by averaging multiple nucleation simulations. The ice nucleation rate *J* is estimated from the volume of water *V* and the nucleation time, *J* = 1/(τ_*J*_ *V*). In the mW model, the volume of water (*V*) is calculated from the molecular volume of ice.^[^ ^37^ ^]^ In this study, 20 independent successful nucleation simulations are used to evaluate the ice nucleation rate. As shown in Figure S9 (Supporting Information), the ice nucleation time and critical nucleus size can be obtained by fitting the MFPT method.

## Conflict of Interest

The authors declare no conflict of interest.

## Supporting information

Supporting Information

## Data Availability

The data that support the findings of this study are available from the corresponding author upon reasonable request.;

## References

[1] K. Jiao , B. Wang , Q. Du , Y. Wang , G. Zhang , Z. Yang , H. Deng , X. Xie , Water and Thermal Management of Proton Exchange Membrane Fuel Cells, Elsevier, Amsterdam 2021.

[2] Y. Sun , C. Xia , B. Yin , H. Gao , J. Han , J. Liu , Int. J. Green Energy 2023, 20, 28.

[3] D. a. Huo , C. M. Hall , Energy and AI 2023, 14, 100289.

[4] Y. Luo , K. Jiao , Prog. Energy Combust. Sci. 2018, 64, 29.

[5] Z. Qin , K. Wu , S. Wu , Q. Du , Y. Yin , B. Wang , S. He , B. Zu , C. Zhang , F. Xi , W. Chen , P. Miao , G. Zhang , J. Gong , K. Jiao , Int. J. Green Energy 2023, 20, 1559.

[6] P. Jiang , Z. Zhan , D. Zhang , C. Wang , H. Zhang , M. Pan , Polymers 2022, 14, 3203. 35956717 10.3390/polym14153203 PMC9370896

[7] T. Giessgen , T. Jahnke , Appl. Energy 2023, 331, 120387.

[8] Y. Zhu , R. Lin , Z. Jiang , D. Zhong , B. Wang , W. Shangguan , L. Han , Int. J. Hydrog. Energy 2019, 44, 7505.

[9] R. Lin , Y. Ren , X. Lin , Z. H. Jiang , Z. Yang , Y. T. Chang , Energy 2017, 123, 367.

[10] J. Tao , X. Wei , P. Ming , X. Wang , S. Jiang , H. Dai , Energy Convers. Manag. 2022, 274, 116465.

[11] Y. Wang , J. Electrochem. Soc. 2017, 154, B1041.

[12] R. J. Balliet , J. Newman , J. Electrochem. Soc. 2011, 158, B927.

[13] G. Gwak , J. Ko , H. Ju , J. Hydrog. Energy 2014, 39, 21927.

[14] T. J. Dursch , G. J. Trigub , R. Lujan , J. F. Liu , R. Mukundan , C. J. Radke , A. Z. Weber , J. Electrochem. Soc. 2014, 161, F199.

[15] P. Oberholzer , P. Boillat , R. Siegrist , R. Perego , A. Kästner , E. Lehmann , G. G. Scherer , A. Wokaun , J. Electrochem. Soc. 2011, 159, B235. 10.1088/0953-8984/23/23/234108 21613688

[16] G. C. Sosso , J. Chen , S. J. Cox , M. Fitzner , P. Pedevilla , A. Zen , A. Michaelides , Chem. Rev. 2016, 116, 7078. 27228560 10.1021/acs.chemrev.5b00744 PMC4919765

[17] B. Glatz , S. Sarupria , Langmuir 2018, 34, 1190. 29020452 10.1021/acs.langmuir.7b02859

[18] L. Lupi , A. Hudait , V. Molinero , J. Am. Chem. Soc. 2014, 136, 3156. 24495074 10.1021/ja411507a

[19] L. Filion , M. Hermes , R. Ni , M. Dijkstra , J. Chem. Phys. 2010, 133, 244115. 21197984 10.1063/1.3506838

[20] T. Li , D. Donadio , G. Russo , G. Gallicd , Phys. Chem. Chem. Phys. 2011, 13, 19807. 21989826 10.1039/c1cp22167a

[21] A. Laio , F. L. Gervasio , Rep. Prog. Phys. 2008, 71, 126601.

[22] N. Giordano , E. Passalacqua , L. Pino , A. Arico , V. Antonucci , M. Vivaldi , K. Kinoshita , Electrochim. Acta 1991, 36, 1979.

[23] C. Zhao , S. Yuan , X. Cheng , Z. Zheng , J. Liu , J. Yin , S. Shen , X. Yan , J. Zhang , Energy and AI 2023, 13, 100245.

[24] S. Wang , Y. Huang , H. Heinz , Sci. Adv. 2021, 7, eabb1435. 34108201 10.1126/sciadv.abb1435 PMC8189588

[25] J.‐C. Dong , M. Su , V. Briega‐Martos , L. Li , J.‐B. Le , P. Radjenovic , X.‐S. Zhou , J. M. Feliu , Z.‐Q. Tian , J.‐F. Li , J. Am. Chem. Soc. 2020, 142, 715. 31887023 10.1021/jacs.9b12803

[26] C. Li , N. C. S. Selvam , J. Fang , Nano‐Micro Lett. 2023, 15, 83. 10.1007/s40820-023-01060-2 PMC10066057 37002489

[27] S. J. Cox , S. M. Kathmann , B. Slater , A. Michaelides , J. Chem. Phys. 2015, 142, 184705. 25978903 10.1063/1.4919715

[28] P. J. Steinhardt , D. R. Nelson , Phys. Rev. B 1983, 28, 784.

[29] A. K. Metya , J. K. Singh , J. Phys. Chem. C 2018, 122, 19056.

[30] Y. Ueki , Y. Tsutsumi , M. Shibahara , Int. J. Heat Mass Transf. 2022, 194, 123004.

[31] T. H. Shen , L. Spillane , J. Peng , Y. Shao‐Horn , V. Tileli , Nat. Catal. 2022, 5, 30. 35141468 10.1038/s41929-021-00723-w PMC8799463

[32] X. X. Zhang , M. Chen , M. Fu , J. Chem. Phys. 2014, 141, 124709. 25273463 10.1063/1.4896149

[33] V. Molinero , E. B. Moore , J. Phys. Chem. B 2009, 113, 4008. 18956896 10.1021/jp805227c

[34] A. P. Thompson , H. M. Aktulg , R. Berger , D. S. Bolintineanu , W. M. Brown , P. S. Crozier , P. J. in ’t Veld , A. Kohlmeyer , S. G. Moore , T. D. Nguyen , R. Shan , M. J. Stevens , J. Tranchida , C. Trott , S. J. Plimpton , Comput. Phys. Commun. 2022, 271, 108171.

[35] E. B. Moore , E. de la Llave , K. Welke , D. A. Scherlis , V. Molinero , Phys. Chem. Chem. Phys. 2010, 12, 4124. 20379503 10.1039/b919724a

[36] J. Wedekind , R. Strey , D. Reguera , J. Chem. Phys. 2007, 126, 134103. 17430012 10.1063/1.2713401

[37] J. C. Johnston , V. Molinero , J. Am. Chem. Soc. 2012, 134, 6650. 22452637 10.1021/ja210878c

