# Supplementary material for: Ice Nucleation Mechanisms on Platinum Surfaces in PEM Fuel Cells: Effects of Surface Morphology and Wettability
Source: Adv Sci (Weinh). 2024 Aug 8;11(38):2406861. doi: 10.1002/advs.202406861 (PMC11481227; doi:10.1002/advs.202406861)
Supplement: Supplementary file 1 — Supporting Information [file ADVS-11-2406861-s001.docx]

**Supplementary Material**

**Ice Nucleation Mechanisms on Platinum Surfaces in PEM Fuel Cells: Effects of Surface Morphology and Wettability**

Jiaqi Wang^1,2^, Linhao Fan^1,2^, Lincai Li^1,2^, Qing Du^1,2*^, Kui Jiao^1,2*^

1 State Key Laboratory of Engines, Tianjin University, 135 Yaguan Road, Tianjin 300350, China

2 National Industry-Education Platform for Energy Storage, Tianjin University, 135 Yaguan Road, Tianjin 300350, China

*Corresponding authors: duqing@tju.edu.cn; [kjiao@tju.edu.cn](mailto:kjiao@tju.edu.cn)

**S1.** **Ice Nucleation Process**


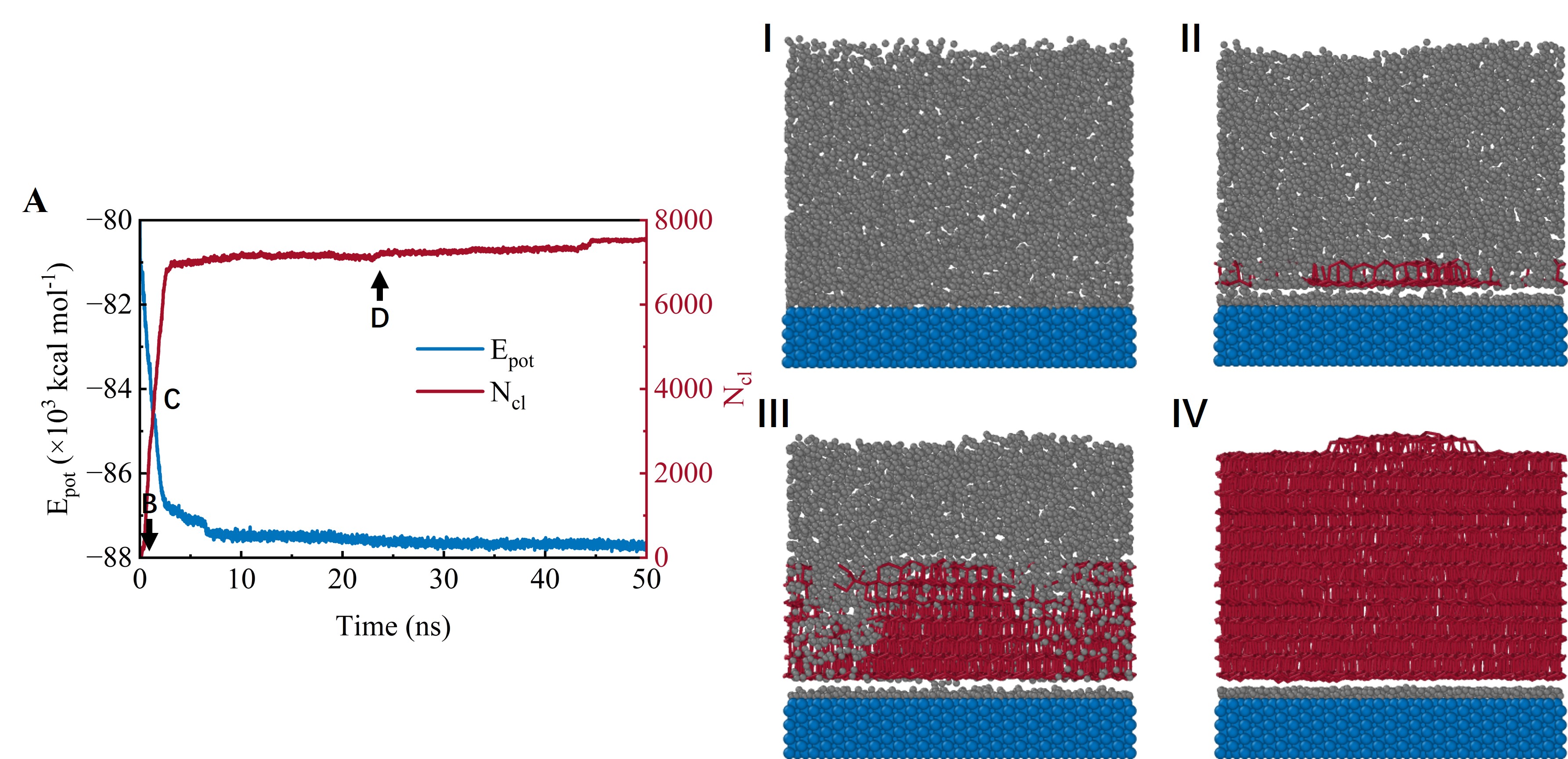


**Figure S1.** Ice nucleation process of supercooled water on Pt(110) surface. (A) Change in the potential energy of water molecules (*E*_pot_) and the number of water molecules in the largest ice cluster (*N*_cl_). (B) Different stages of ice cluster formation during simulated ice crystallization. The ice cluster is represented by red rods, while the mW molecules are represented by gray balls.


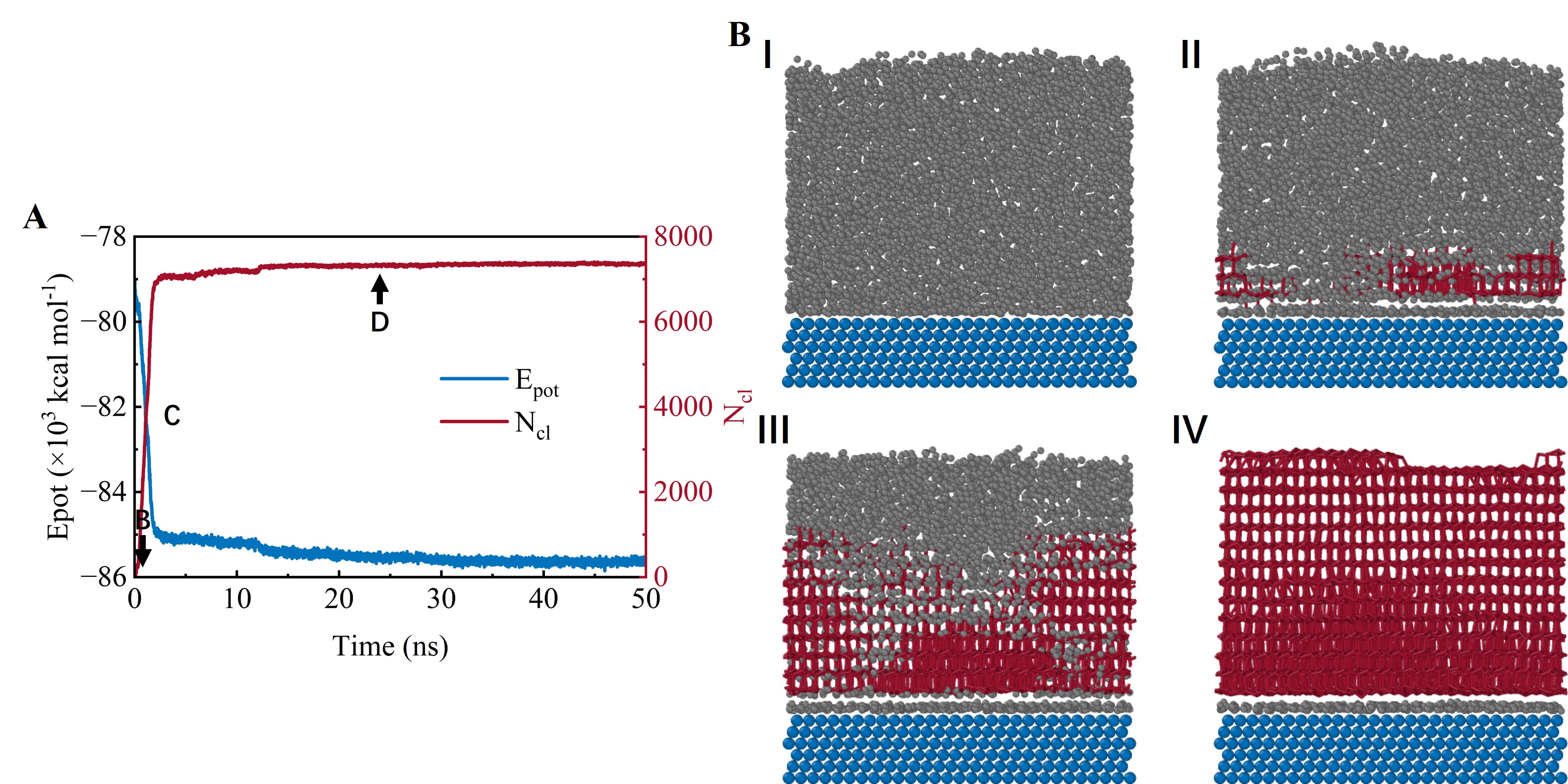


**Figure S2.** Ice nucleation process of supercooled water on Pt(111) surface. (A) Change in the potential energy of water molecules (*E*_pot_) and the number of water molecules in the largest ice cluster (*N*_cl_). (B) Different stages of ice cluster formation during simulated ice crystallization. The ice cluster is represented by red rods, while the mW molecules are represented by gray balls.


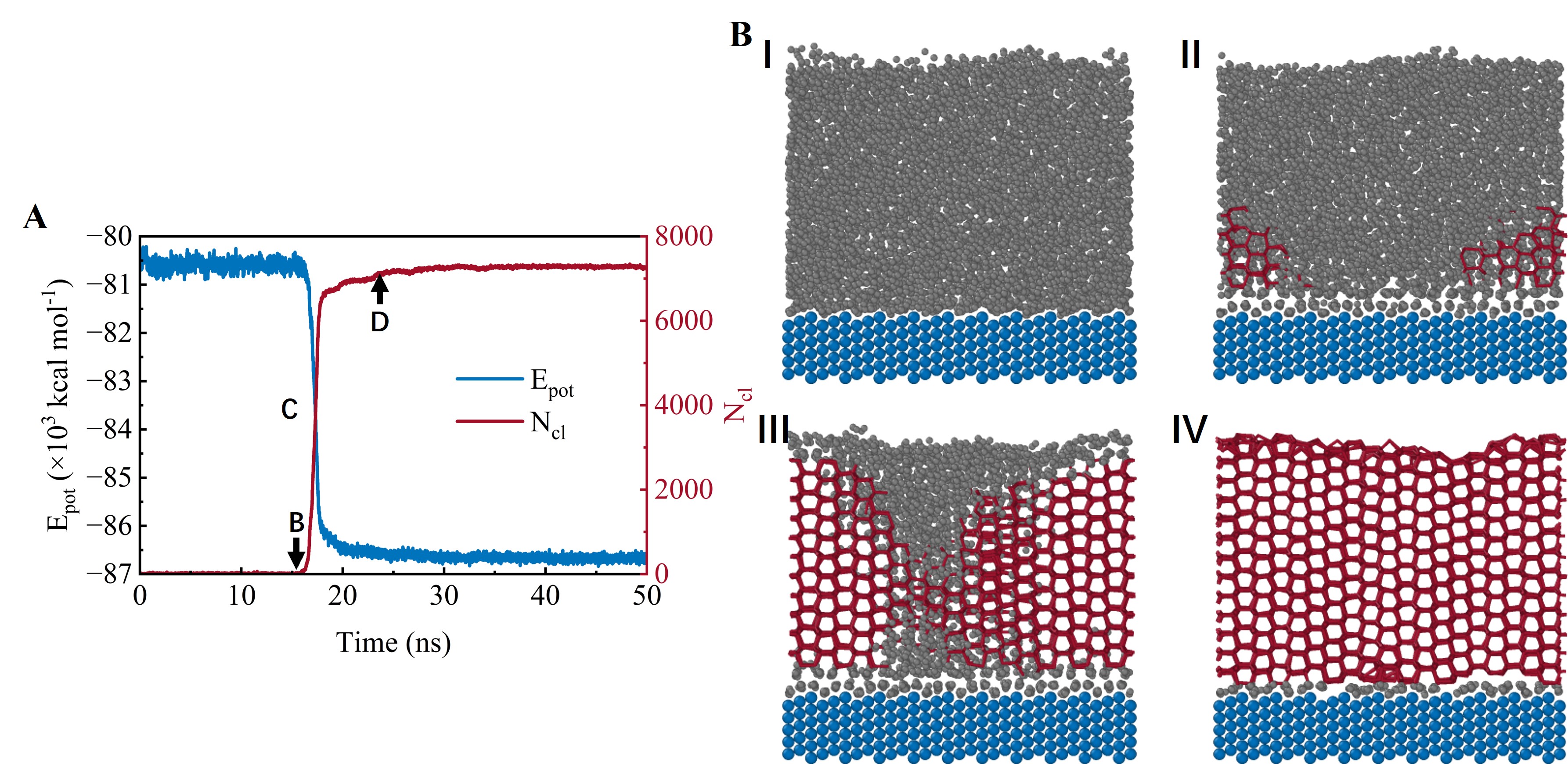


**Figure S3.** Ice nucleation process of supercooled water on Pt(211) surface. (A) Change in the potential energy of water molecules (*E*_pot_) and the number of water molecules in the largest ice cluster (*N*_cl_). (B) Different stages of ice cluster formation during simulated ice crystallization. The ice cluster is represented by red rods, while the mW molecules are represented by gray balls.

**S2. Distribution of Water Number Density**


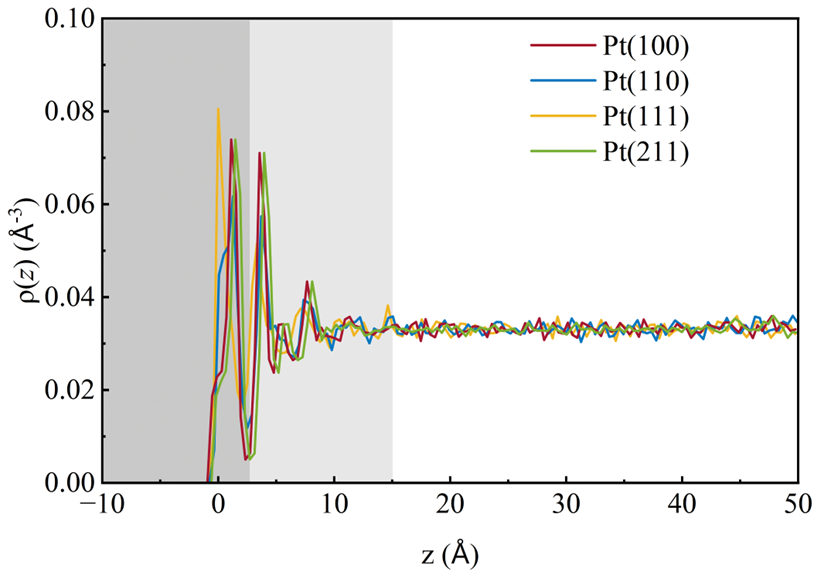


**Figure S4.** Distribution of water number density on Pt surfaces.

**S3. Cubic Ice and Hexagonal Ice Distribution**


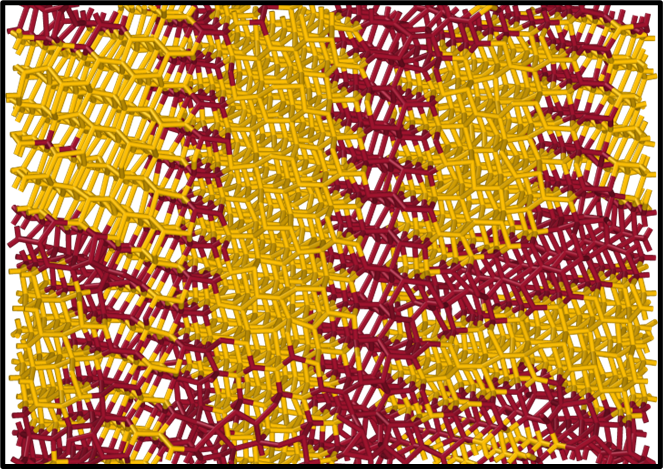


**Figure S5.** Comparison of the distribution of cubic ice and hexagonal ice of homogeneous ice nucleation.

**S4.** **Contact Angles on Pt Surfaces**


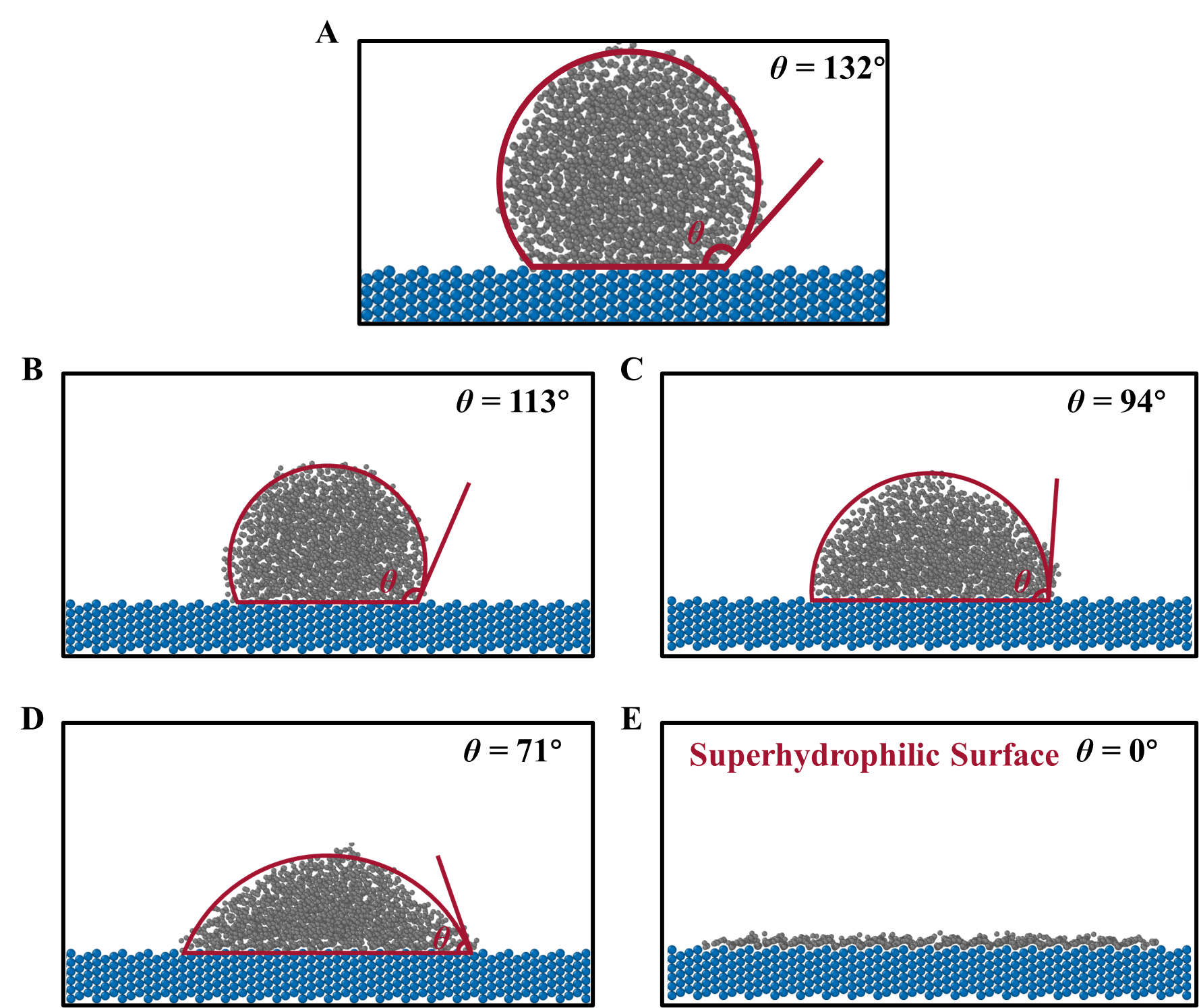


**Figure S6.** Contact angles on Pt(211) surfaces with different wettability.

**S5. Ice Nucleation on Hydrophobic Surface**


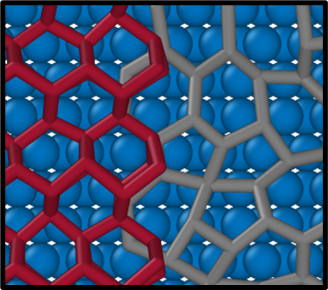


**Figure S7.** The top view of mW molecules on the Pt(211) with the contact angles of 132° after freezing. In all cases, the second layer is red, while the contact layer is gray. Part of the overlayer and second layer are shown.

**S6.** **Distribution of the q_6_ Order** **Parameter**


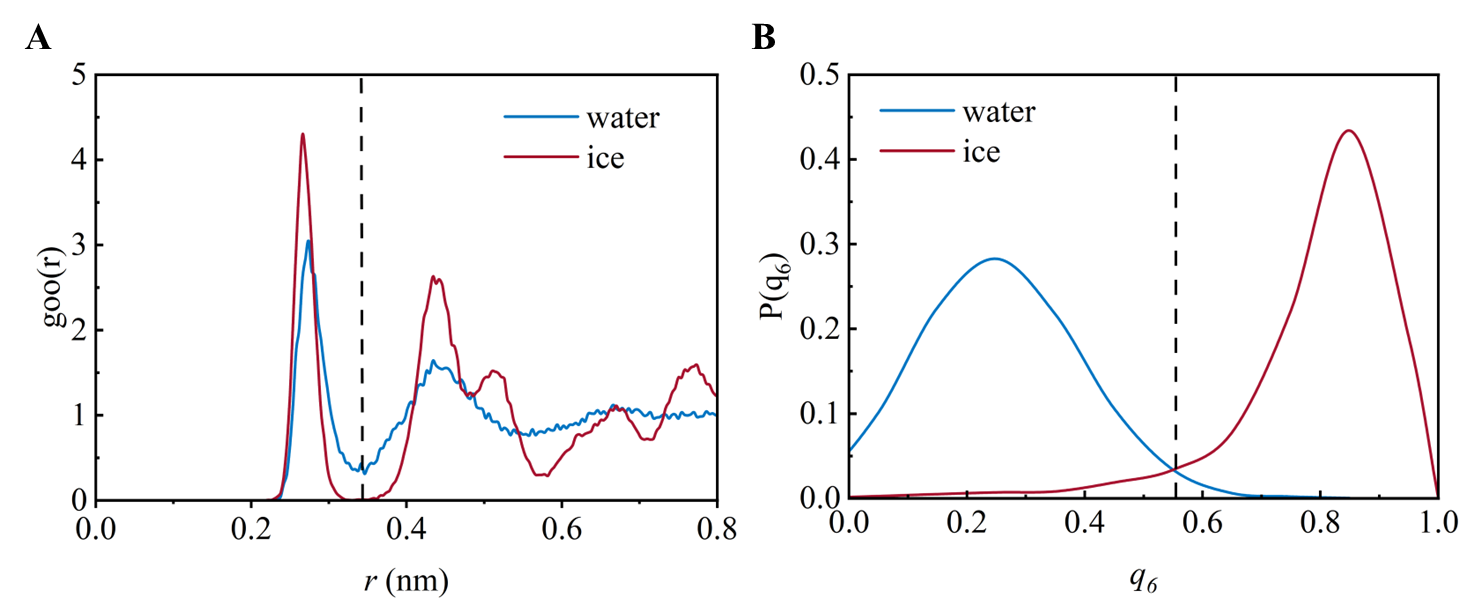


**Figure S8.** (A) Radial distribution function (RDF) of water and ice. (B) The distribution of *q_6_* parameters of water and ice.

Nucleation was monitored by following the change in the potential energy. Moreover, the number of molecules in the largest ice-like cluster, *N_cl_*, is monitored. To accurately identify ice-like molecules, the water molecule local order parameter *q_6_* are calculated by simulating trajectories. The radial distribution function (RDF) of water and ice is first calculated, as shown in Fig. S8A. The results shows that the first coordination shell of water and ice appear when the distance *r* is 0.35 nm. Since the *q_6_* can measure the order degree of atoms in the first coordination shell, the 0.35 nm is used as cutoff to calculate the distribution of *q_6_*. As shown in Fig. S8B, *q_6_* is a good parameter to differentiate water and ice. Specifically, the 0.58 of *q_6_* is chosen as cutoff to identify ice-like molecules.

**S7.** **Nucleation Rate: Mean first-passage time (MFPT) method**


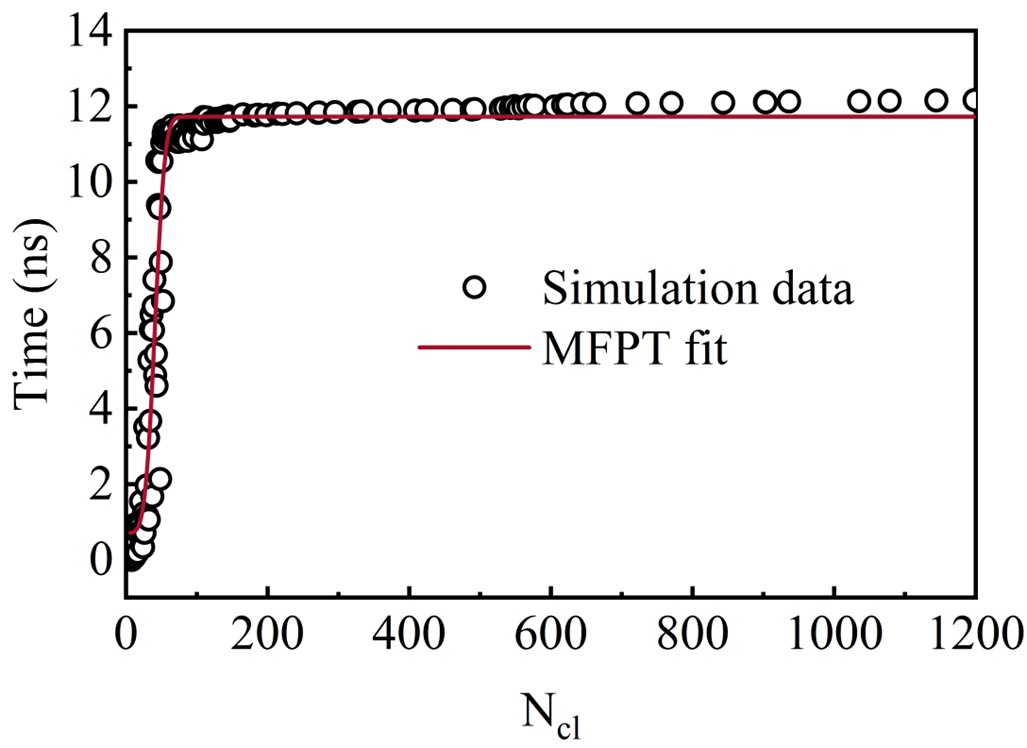


**Figure S9.** Plot of the MFPT curve for water icing on Pt surface. *N_cl_* is the number of water molecules in the largest ice cluster.

The the mean first passage time (MFPT) method proposed by Wedekind et al [1] is used to evaluate the ice nucleation rate (J) The MFPT method can directly provide the nucleation time and the size of the critical nuclei by fitting the MFPT curve using the following expression:

$\tau(n)=\frac{\tau_{J}}{2}\left\{1+erf\left[\left(n-n^{*}\right)c\right]\right\}$ (S1)

where $\tau_{J}$ and *n^*^* are the critical nucleation time and the size of the critical nucleus, respectively. The parameter *c* is a constant associated with the Zeldovich factor Z, $c=Z\sqrt{\pi}$. The nucleation time for each cluster of size is obtained by averaging multiple nucleation simulations. The Fig. S9 shows the MFPT as a function of cluster size obtained from MD simulations and the corresponding fit to Eq. (S1). The nucleation rate J is estimated from the volume of water V and the nucleation time, $J=1/(\tau_{J}V)$.

**References**

1. J. Wedekind, R. Strey and D. Reguera, *J. Chem. Phys.*, 2007, **126**, 134103.
